# Hyperpolarization by activation of halorhodopsin results in enhanced synaptic transmission: Neuromuscular junction and CNS circuit

**DOI:** 10.1371/journal.pone.0200107

**Published:** 2018-07-03

**Authors:** Matthew Mattingly, Kristin Weineck, Jennifer Costa, Robin L. Cooper

**Affiliations:** 1 Department of Biology and Center for Muscle Biology, University of Kentucky, Lexington, Kentucky, United States of America; 2 Goethe University Frankfurt am Main, Frankfurt, Germany; EPFL, SWITZERLAND

## Abstract

Optogenetics offers a unique method to regulate the activity of select neural circuits. However, the electrophysiological consequences of targeted optogenetic manipulation upon the entire circuit remain poorly understood. Analysis of the sensory-CNS-motor circuit in *Drosophil*a larvae expressing eHpHR and ChR2-XXL revealed unexpected patterns of excitability. Optical stimulation of motor neurons targeted to express eNpHR resulted in inhibition followed by excitation of body wall contraction with repetitive stimulation in intact larvae. In situ preparations with direct electrophysiological measures showed an increased responsiveness to excitatory synaptic activity induced by sensory stimulation within a functional neural circuit. To ensure proper function of eNpHR and ChR2-XXL they were expressed in body wall muscle and direct electrophysiological measurements were obtained. Under eNpHR induced hyperpolarization the muscle remained excitable with increased amplitude of excitatory postsynaptic synaptic potentials. Theoretical models to explain the observations are presented. This study aids in increasing the understanding of the varied possible influences with light activated proteins within intact neural circuits.

## Introduction

The ability to alter selective neural circuits at various times with high temporal resolution is now possible with a variety of optical techniques. However, being able to finely tune defined circuits is still challenging but strides are being made for therapeutic applications and adjustments in behavior [1–3]. Recent approaches are advancing in the use of flexible and very thin optic fibers to control neurons in deep regions of the CNS in model animals by the use of light activated proteins (i.e., optogenetics) with good spatial and temporal resolution [4–8].

To measure synaptic efficacy within neural circuits, direct measures of the postsynaptic target’s membrane potential and frequency of activity are needed. Both can be relatively hard to measure with imaging techniques. Imaging with fMRI and ionic indicators (Ca^2+^, Na^+^) provides information about location and which neurons are active but not to the degree of quantifying synaptic communication reliably.

Combining the ability to optically stimulate neurons while making direct measures advances this goal [9]. However, the interference of light on electrical monitoring devices can be of concern [10]. Direct electrophysiological measures across cell membranes are feasible in model systems for drawing inference to more complex systems such as the human brain. Bridging optogenetic control of cellular activity and the direct measurement of synaptic responses provides a clear understanding of how intact circuits are functioning and if the experimental paradigms with an optogenetic drive are producing the desired outcomes [8,11].

Understanding the effects on synaptic output when driving inputs, which maybe a few neurons directly removed within an intact neural circuit, is complex due to various cellular responses from graded excitation and inhibition. In addition, the timing of acute (seconds to minutes) and longer term (minutes to hours) modifications such as with short term facilitation, long-term potentiation, volume transmission and hormonal modulation can make detecting the precise mechanisms behind synaptic structural changes challenging. Modifying neural activity over developmental periods or in disease states is an interest for several therapeutic applications. Thus, addressing the impact of repetitive use and fine control of light activated channels in modifying neural circuits is of interest. The power of optogenetics has provided an amazing ability to direct expression of light sensitive proteins while observing the effects on behavior and monitoring circuit formation over the long term [12–19]. Manipulation of the expression of non-native light activated proteins has recently been applied in clinical human treatments and will likely increase in use in humans and potentially during development and repair of the nervous system [16,20–24]. Commonly used light activated proteins for exciting and inhibiting cells are channelrhodopsin (variants which can conduct Na^+^ and Ca^2+^ ions), chloride-conducting channelrhodopsins (iC1C2), Archaerhodopsin (outward proton pump) and halorhodopsin (eNpHR a chloride pump used to inhibit neural activity) [25–27]. However, there are also limitations with the use of these proteins in reproducibility and expression. [7,10,28–32] These concerns are now being addressed in functional circuits with direct electrical measures of synaptic function [8,10,33,34].

In being able to acutely excite cells with channelrhodopsins and the variant forms to varying degrees of sensitivity to light and pH on ionic flux have been examined [28,35–37]. Prolonged excitation of the light sensitive cation ion channels or even over expression of proteins and fluorescent markers may lead to cellular damage [10,38]. Undesirable effects may occur with short light pulses of five to ten seconds such as depolarization inactivation of voltage-gated Na^+^ channel [33,34,39]. This can lead to cellular inhibition of a physiological response to normal synaptic inputs within a neural circuit [34]. However, such light pulses of five to ten seconds may be desirable to insure activation of the cells in intact larva or adult stages of insects with thicker cuticle or for controlling a complex brain from a seizure. Similarly, the ability to directly hyperpolarize a cell with the use of halorhodopsin or a Cl^-^ channel opsin may induce undesirable effects.

Since halorhodopsin is a Cl^-^ ion pump, activating this protein does not necessarily decrease excitatory input on the same cell as would a Cl^-^ channel such as with presynaptic inhibition [40] or direct inhibition by GABA-A receptors. Instead, hyperpolarization can allow a cell to be more excitable by lowering the threshold for excitation by removing voltage-gated Na^+^ channel inactivation [41,42].

In studies using *Drosophila* larval with halorhodopsin (eNpHR) expressed in motor neurons, it was shown the larvae relax with activating light but sometimes contract after the activating light is turned off [39]. This was attributed to be due to post-inhibitory rebound of the motor neurons. In the study herein, we demonstrate that synaptic excitatory depolarization increased in amplitude during halorhodopsin activation and a rebound neural circuit activity increased. The responses may well be cell dependent within neural tissue in a given animal due to differences in neuronal cell types. In some types of neurons as many as 10% of the voltage gated channels are in an inactive state at rest [41,43–45]. Thus, hyperpolarizing a cell may remove the Na^+^ channel inactivation maybe removed lowering the spiking threshold.

Generally, in reducing the resting membrane potential of a target cell the driving gradient for Na^+^ and Ca^2+^ is increased. Thus, synaptic responses which arise from glutamate or acetylcholine ionotropic receptors on dendrites can be enhanced. This is not only the case for evoked synaptic events but also for spontaneous quantal events as the driving gradient for the excitatory receptor potentials will also be increased. Since spontaneous quantal events are presumed to have a role as a regulator of homeostatic plasticity [46], altering the frequency or potentially the amplitude of events may have an impact on synaptic development and homeostasis which could impact evoked transmission. During development the fine tuning in evoked and spontaneous synaptic activity is crucial for proper formation of neural circuits.

The larval *Drosophila* neuromuscular junction (NMJ) serves as a model preparation to address synaptic function and plasticity [47–51]. A sensory-CNS-motor neuron circuit in the larval system also provides a measure of central synaptic function which is readily related to behavior in the intact animal [14,52,53]. These two models were used in this study to address the consequences in manipulating the activity profile of defined neurons and the developmental impacts on the formation of neural circuits. This study highlights the importance of conducting electrophysiological studies in addition to behavioral studies for one’s understanding in long term use of these light activated proteins and conceptual understanding in controlling neuronal circuits.

## Materials and methods

### Drosophila lines

The first filial 1 (F1) generations were obtained by crossing virgin females of a halorhodopsin line, w*;P{UAS-eNpHR-YFP} (BDSC# 41752) or y^1^ w^1118^; PBac{UAS-ChR2.XXL}VK00018 (BDSC stock # 58374) with males of w*;P{D42-Gal4} (BDSC # 8816) or w;Kr/CyO;Mef2-Gal4,UAS-mCD8.RFP. The D42-Gal4 line was used specifically for its high level of expression in motor neurons [54,55]. Myocyte enhancer factor-2 (MEF2) plays an essential role at the embryonic stage of muscle development and the GAL4-Mef2 line was used to express the opsins in skeletal muscle (kindly provided by Dr. Doug Harrison, University of Kentucky). The larvae expressing RFP (red) in muscle were selected for studies using muscle.

### Fly rearing

All parental lines used were held for a few days at 22°C in a 12-hour light/dark incubator before being crossed. All animals were maintained in vials partially filled with a cornmeal-agar-dextrose-yeast medium. The general maintenance is described in [56]. After crossing lines, the larvae were raised in complete darkness.

### Preparation of fly food with ATR supplement

All trans-retinal (ATR; Sigma-Aldrich, St. Louis, MO, USA) was diluted in standard fly food to a final concentration of 1 mM (for ChR2 use) or 10 mM (for eNpHr use) and protected from light with aluminum foil. For control experiments, larvae were cultured in food that only contained the solvent (absolute ethanol in the same volume used for the ATR mixtures in the fly food). The ATR or ethanol food mixtures were left with a cotton plug at 4°C to allow evaporation of the alcohol solvent from the mixture. Adult flies from the driver (Gal4) lines and the UAS-ChR2 and UAS-eNpHR effector lines were crossed on standard fly food. After the cross, early 2^nd^ instar larvae were removed from standard food vials and placed in ATR-food mixtures and left for 48 hours prior to testing. It has been noted that larval development slows in the presence of ethanol, so precautions were taken to limit its developmental influence by evaporating the alcohol.

### Behavior

Locomotor behavior was viewed by placing larvae on a plastic Petri dish with etching on the surface to provide grip for the larvae. A small coating of apple juice was rubbed on the surface and larvae are placed on the dish with lid covering the dish [14]. The larvae were left for one minute to acclimate to their new environment. Body wall movements were recorded while being exposed to a dim white light or a darkroom red light and when exposed to diffuse blue or yellow light from an LED mounted below the stage [34,57]. Larvae were exposed to blue and yellow light in 15 sec bursts, followed by a 30 min recovery period under dim white, or red light. Dim red light was given by using white light fitted with a white light filter (Edmond Scientific, cD43,951). The locomotion activities were recorded with a webcam (WEBCAM HD4110, Hewlett-Packard Company, Palo Alto, CA) and stored on a computer. The activity was recorded at 25 frames per second for various experimental paradigms (see Results). Movies to illustrate the larvae movements were made with IR camera in which the camera provided the IR light source (see supplemental data). The webcam was able to monitor the larvae during the LED light pulses whereas the IR camera becomes saturated by the bright LEDs but can record behaviors well with minimal white light.

### Intracellular recordings from the neuromuscular junction

Larval dissections were performed as described previously [58,59]. In brief, the preparations were “fileted” along the mid-dorsal longitudinal axis and pinned flat. Excitatory postsynaptic potentials (EPSPs) were evoked by exposing the dissected preparations with blue light (470nm wavelength, LEDsupply, LXML-PB01-0040) or yellow-lime light (567.5 nM wavelength, LEDsupply, LXML-PM02-0000) from a high intensity LED that was focused on the specimen through a 10x ocular objective while the EPSPs were measured [53,57]. Intracellular recordings from muscle 6 were made with microelectrodes filled with 3M KCl having a resistance of 30–60 MΩ. Responses were recorded with a 1X LU head stage and an Axoclamp 2A amplifier. Electrical signals were recorded to a computer A/D interface (ADInstruments). All events were measured and calibrated with the LabChart7 software (ADInstruments). All experiments were performed at room temperature (21–22°C). The larval preparations were dissected, fileted, and bathed in a physiological saline and the responses in the presence of dim white light, followed by blue light or yellow light pulses to detect the response in the muscle following development in complete darkness. A modified HL3 saline (NaCl 70mM, KCl 5mM, MgCl_2_.6H_2_O 20mM, NaHCO_3_ 10mM, Trehalose 5mM, sucrose 115mM, Trizma acid 25mM or BES 25mM, and CaCl_2_.2H_2_O 1mM, the pH 7.1; [60]) was used as a physiological saline. The suction electrodes which were used to stimulate the segmental nerves in each preparation was slightly different; therefore, a range of stimulation voltages were used. Depending on how tight the seal is with the nerve, the voltage must be adjusted to evoked action potentials in the sensory nerves. The current is not directly varied but reflects the voltage and the seal resistance for each preparation. By transecting segmental roots close to muscle fibers on one side of the larvae, after it is opened and pinned out, the sensory neurons provide input into the CNS under the control of the external stimulator. Pulse trains of 5 to 10 stimuli given in a range of 40 to 60 Hz are determined until a series of motor neurons are recruited on the contralateral side of the sensory neurons (described in [52]). Recordings of the EPSPs in muscle m6 reflect the frequency of the motor nerve activity to the muscle. Thus, a sensory-CNS-motor neuron circuit is driven from one hemisegment to another. This is very similar to an intact larva being touched on the lateral side of the body wall and the larvae bending or speeding up in locomotion in response to the stimuli [61]. In recording spontaneous quantal events the segmental nerves are not cut, and the CNS was left intact. In cases when only spontaneous vesicle fusion events were recorded in the muscle fibers the motor neurons were not electrically stimulated.

### Statistical analysis

SigmaPlot (version 13.0) was used for graphing and statistical analysis. For behavioral analysis a sign rank test was used with a confidence level of P ≤0.05 is considered statistically significant. The electrophysiological analysis is presented as percent change from control (without ATR feeding or parental lines not expressing opsins). There was considerable variation among baseline EPSP frequency from preparation to preparation in the sensory-CNS-motor neuron activation so a before and during as well as after the light activation of opsins was used for assessment.

## Results

### Intact larval behavior

**Fig 1:**
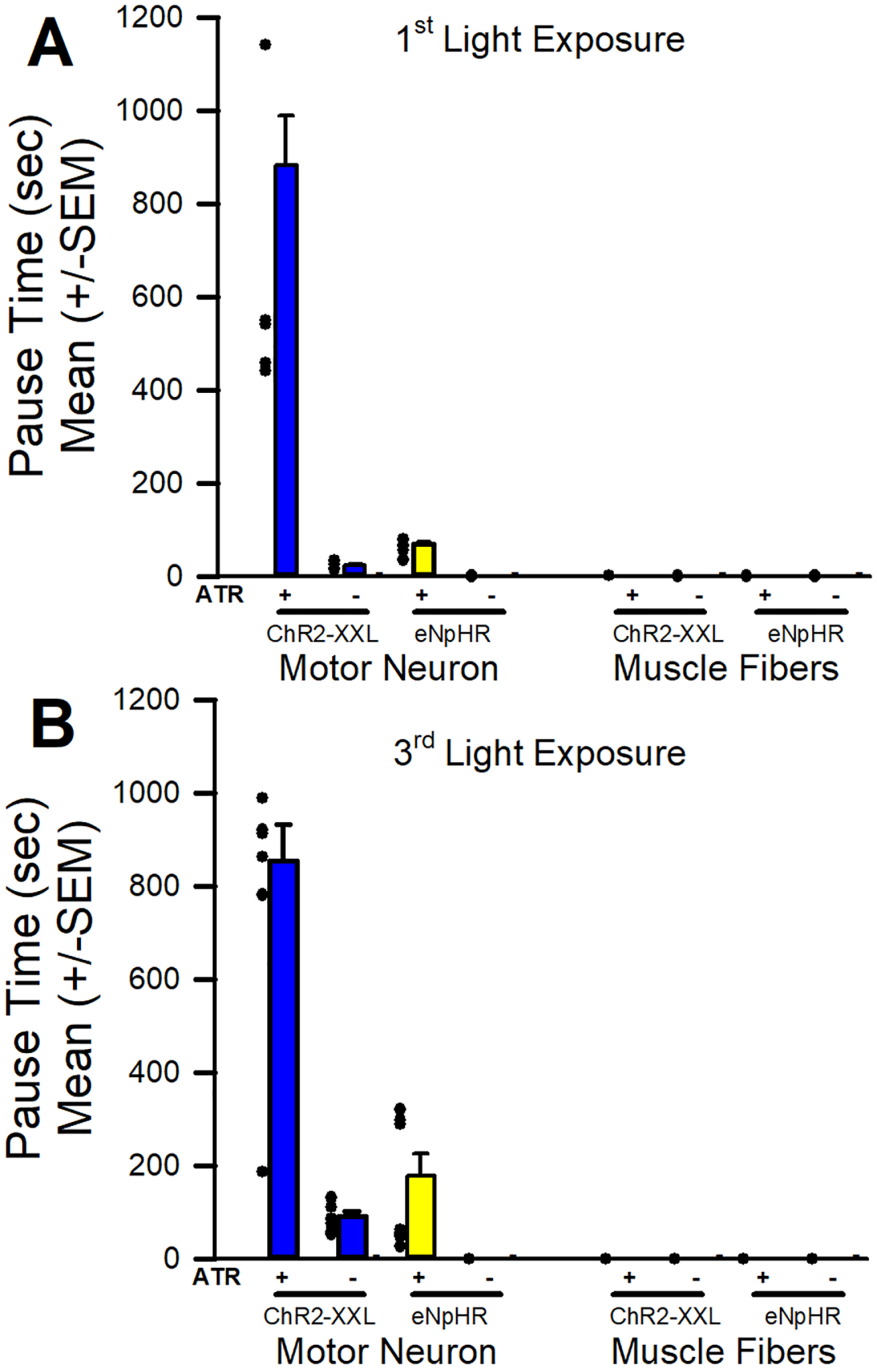
The time taken before larvae started to crawl with a full body wall contraction after activation of ChR2-XXL or eNpHR when expressed in motor neurons or muscle. Three repetitive trials of 15 sec of light exposure followed with 30 min dim red light were given. The time taken in the pause of movement from when the light was turned off to the 1st movement is graphed for individual larvae as well as the mean (+/- SEM) after the 1st light exposure and the 3rd light exposure with the series. Note that the 1st light exposure resulted in the larvae relaxing and becoming flaccid but with subsequent light exposures the larvae were contracted for the motor neurons expressing eNpHR. In both conditions, after the yellow light was turned off, the larvae pause before being able to crawl again.

We found the commonly used 1% agar apple juice plates presented some problems when larvae burrow into the agar as one cannot tell if the larvae stop crawling or if the animal was burrowing. In past studies, we did use agar dishes but now use this modified approach with etching the bottom of the plastic dish to provide a gripping surface for the larvae. The ChR2-XXL expressed in motor neurons is very sensitive to white light, even without supplementing the food with ATR. For good behavioral responses when animals are covered with food for long term culturing, this line proved to be consistent for the extra sensitivity through the media. Also, to compare results to a previous study [34] we used this sensitive line for the current study. However, we found using a standard darkroom red light source (Edmond Scientific, cD43,951 filter) did not produce behavioral responses in the ChR2-XXL x D42 larvae fed or not fed ATR. Upon blue light exposure full body contraction was apparent. An IR light source and IR sensitive camera to illustrate larval behavioral responses proved to be superior to the standard darkroom red light source. The supplemental movies are recorded with IR camera. As expected for activation of ChR2-XXL expressed in motor neurons and muscle a rapid contraction occurs (S1 Video). The time of recovery from the ChR2-XXL activation was much longer for motor neuron activation as compared to body wall muscles (Fig 1). Activation of ChR2-XXL expressed in the muscle causes the larvae to contract during the blue light pulse, but they regain body wall movements rapidly after the light is turned off. Of key interest is the result of eNpHR activation when expressed in motor neurons and muscle (see S2 and S3 Videos as illustrations). We used three 15 sec pulses of yellow light with 30 min of rest between the light stimuli. The pause time after the light was turned off until larvae started the 1st full body wall contraction to produce and inch worm type of locomotion is shown (Fig 1). Since we observed some differences in the delay of moments with the trials over time from the 1st light pulse (Fig 1A) to the 3rd pulse (Fig 1B), the delays are graphed separately. As Inada et al., [39] observed and we confirmed, larvae relax with the initial 15 sec yellow light pulse but we found following the cessation of light an inactivity of the larvae for a period of time. We also repeated the stimulus trail two more times after 30 min of rest in between trials and the subsequent light pulses resulted in contraction of the larvae with a period of paralysis which was not as long as the 1^st^ exposure. The larvae showed a slight reduction of movement without pausing by the 3rd light pulse with eNpHR activation in muscle fibers.

### Motor neurons expressing ChR2-XXL or eNpHr while evoking sensory-motor circuit

**Fig 2:**
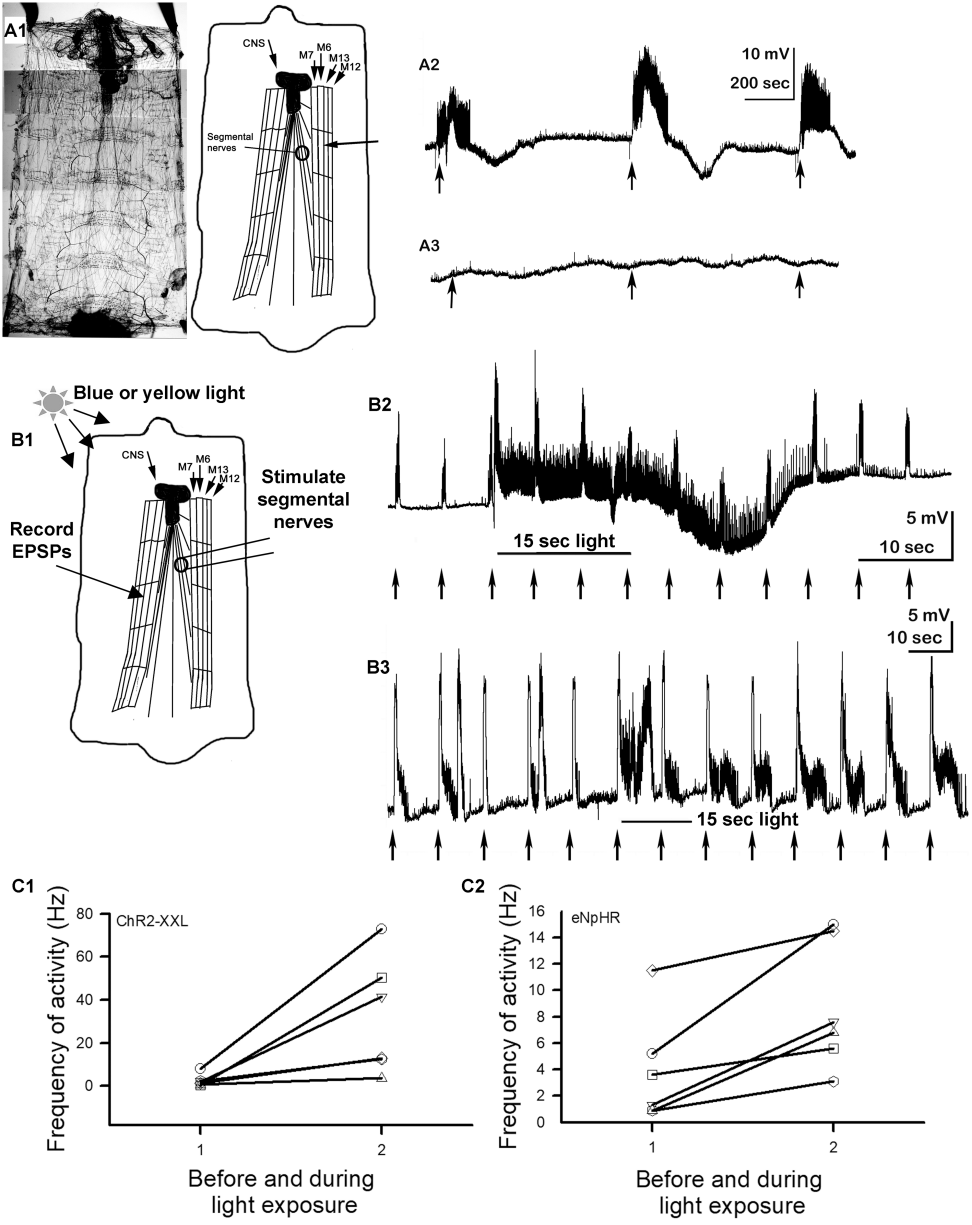
The activation of ChR2-XXL or eNpHR expressed in motor neurons without and with evoking the sensory-CNS-motor circuit. (A) In the absence of stimulating the sensory nerves, (A2) exciting with blue light evokes EPSPs in the muscle fibers. (A3) When eNpHR is ChR2-XXL expressed in motor nerves expressed in motor nerves and activated with yellow light no EPSPs are evoked in the muscle fibers. (B) In the presence of evoking the sensory-CNS-motor circuit and exciting (B2) ChR2-XXL or (B3) eNpHR there is an increased responsiveness of the motor nerve with an increased firing frequency during and sometimes after the light stimulus is turned off. The downward movement of the trace in B2 is a muscle movement artifact from the intense stimulation and not hyperpolarization of the muscle fiber. Each small arrow indicates a 15 sec light pulse in A2 and A3 and an evoked sensory-CNS-motor stimulus in B2 and B3. To evoke the sensory-CNS-motor circuit using an in-situ Drosophila larva, the segmental nerve or nerves on one or two caudal segments is transected and stimulated to evoke contralateral motor neurons. The evoked excitatory postsynaptic potentials (EPSPs), which are graded in larval muscles, are recorded in muscle 6 in segment 3 of 4. The frequency of the evoked EPSPs in the evoked stimulus trail prior to the light exposure and the first trail during light exposure were used as a measure of the alteration in evoking the motor neuron activity for ChR2-XXL (C1) or eNpHR (C2) expressed in motor neurons for each of the 6 preparations for each condition.

As expected, activation of ChR2-XXL expressed in motor neurons caused the motor neurons to be electrically excited resulting in EPSPs in the muscle with the absence of a sensory drive (Fig 2A1 and 2A2). This is repeatable within a preparation although the frequency of the bursts in activity varies. All six preparations examined resulted in this bursting behavior (N = 6, P<0.05 sign test). In motor neurons expressing eNpHR no differences occurred in the muscle when exposing the preparations to yellow light. The lack in inducing any activity occurred consistently in six preparations (N = 6, P<0.05 sign test). One may have expected fewer miniature synaptic responses from random vesicle fusion if the nerve terminal is hyperpolarized and there is some relation to membrane potential to the polarized membrane charge interacting with vesicle docking proteins [62]. More likely one might expect some rebound excitation of the motor nerve terminal after the light is turned off resulting in a depolarized terminal and evoking vesicle fusion as known to occur with mammalian neurons [63,64,65]. The concept is that the inactivation of T-type calcium channels at rest is removed upon hyperpolarization. Upon returning to the resting membrane potential the channels are activated enough to produce rebound action potentials. This may not be the case in the motor terminals at these NMJs since no EPSPs occur in relation to the light only exposure for 15 seconds (Fig 2A3, arrows).

To understand the influence on the neural circuitry when exciting or depressing motor neurons through light activated channels, an in-situ paradigm which integrates sensory input was used [52]. This sensory drive normally evokes muscular movements for obtaining coordinated movements in the intact larvae. In this case, the segmental roots drive a CNS response to recruit motor neurons which is manifested as EPSPs in identifiable muscles as shown in Fig 2B1 and 2B2. The paradigm drives sensory input by electrical stimulation which is analogous to touching the larvae on the caudal midline and produces relatively consistent responses. Tweaking of the stimulus is needed as not to elicit a large volley of motor activity but just enough to evoke motor units beyond each short stimulus train. The reason not to overdrive the sensory input is to soothe evoked activity between each pulse train so that an increase or a decrease in the evoked responses can be readily detected. Evoked sensory to motor drive is readily observed in Fig 2B2 for each pulse train before, during and after light exposure which activated the motor units with the sensory drive (arrows shown). The evoked neural circuit, driven by electrical stimulation of sensory nerves (arrows in Fig 2B2), was altered during ChR2-XXL light activation when expressed in motor neurons in all preparations examined (Fig 2C1; N = 6, P<0.05 sign test). The light induced enhancement of sensory driven recruitment was enhanced even after the light activation of ChR2-XXL was stopped. When the motor neurons expressing eNpHR are activated with light, while sensory drive was stimulated, evoked motor units were recruited (Fig 2B3, first five arrows). However, during the exposure to 15 sec of yellow light the motor units were not only more active during the light exposure but even after the light was not present the units were electrically driven with enhanced sensitivity (Fig 2B3, last six arrows shown). This enhanced activity was present in all six preparations examined Fig 2C2 (N = 6, P<0.05 sign test). Of particular interest is the much-heightened response during 15 sec yellow light exposures while evoking the sensory drive. One would have generally assumed the evoked synaptic drive onto the hyperpolarized motor neurons to be potentially suppressed.

### Skeletal muscle expressing ChR2-XXL or eNpHR effect on synaptic function

**Fig 3:**
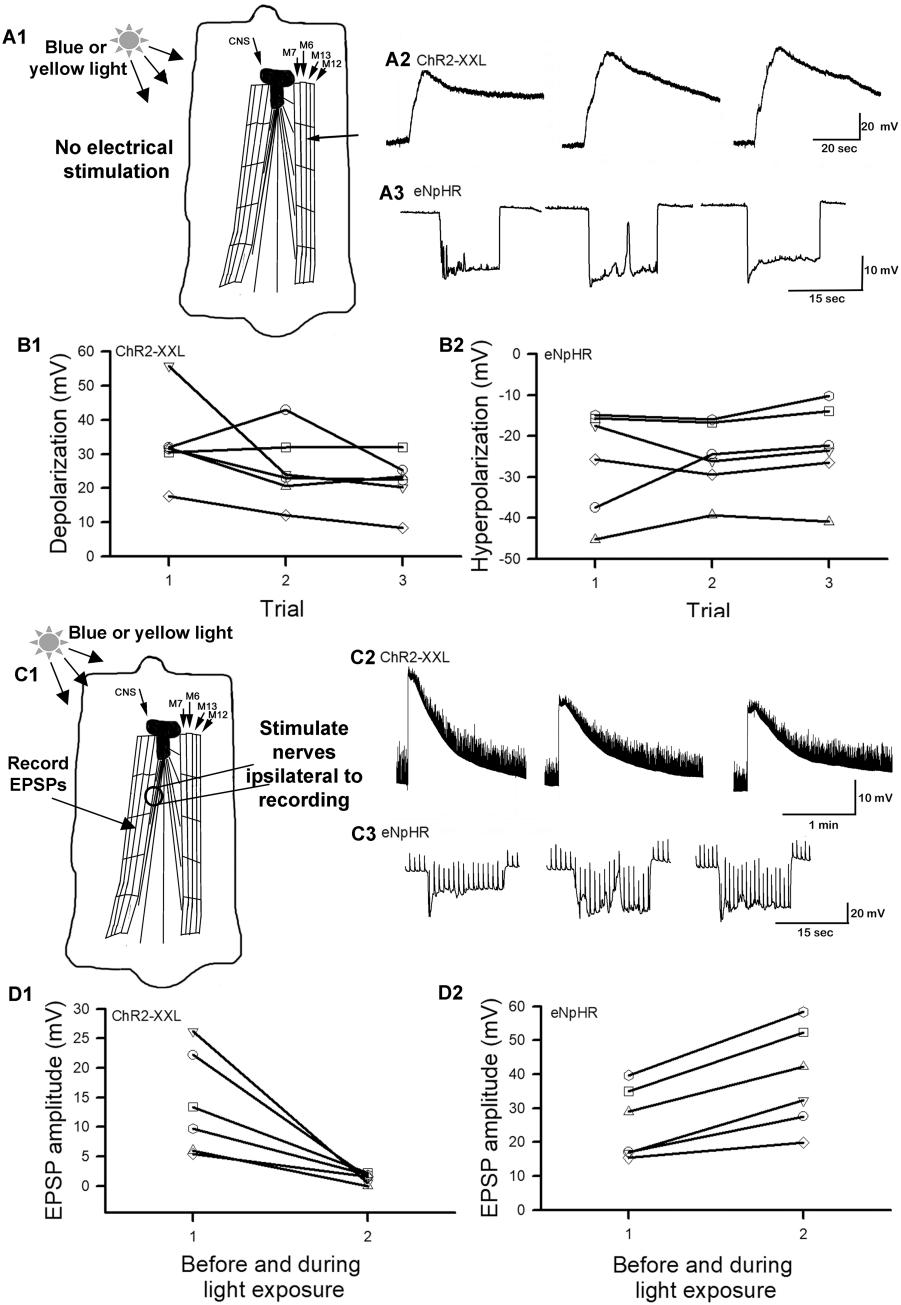
The activation of ChR2-XXL or eNpHR expressed in muscle fibers without and with directly evoking the motor nerves to the muscle fibers. (A1) In the absence of stimulating the motor nerve, (A2) exciting ChR2-XXL expressed in muscle with blue light depolarizes the muscle fibers even longer than the light stimulus. (A3) When eNpHR is expressed in muscle and activated with yellow light the muscle fibers hyperpolarize and following tightly the temporal activation of the eNpHR. The cause of the short depolarization peak during the hyperpolarization within the 2^nd^ trial shown in A3 is unknown but perhaps due to a pause in the pump activity or a spontaneously evoked synaptic response. The peak amplitude in depolarization for ChR2-XXL and peak hyperpolarization for eNpHR in the three repetitive trails, with 10 minutes of dark, are shown (B1 and B2, respectively). (C1) In the presence of evoking the motor nerve directly with a stimulating electrode, delivering continuous stimuli at 0.5 Hz, and exciting (C2) ChR2-XXL there is an decrease in the absolute amplitude of the EPSPs. (C3) However, when eNpHR is excited during the 0.5 Hz stimulation of the motor nerve, there is an increase in the absolute EPSP amplitude. The muscle serves as a model of what could be occurring in the dendrites of the motor neurons during synaptic activity and excitation of ChR2-XXL or eNpHR expressed in the motor neurons. The changes in the evoked EPSP before and during light exposure for ChR2-XXL (D1) or eNpHR (D2) are shown for 6 preparations.

The unexpected excitation of motor neurons during eNpHR activation, necessitated testing to ensure the ChR2-XXL and eNpHR channels were performing as expected. For this analysis the effects of light activation on the channels while expressed in muscle fibers were examined. While monitoring the membrane potential in m6 during blue light excitation of ChR2-XXL for 15 sec the muscle rapidly depolarized and slowly recovered (Fig 3A1 and 3A2). The depolarizations were repeatable with 10 minute rest periods between light pulses, although there was variation among some preparations in providing a consistent amount of depolarization with each trial (Fig 3B1). All six preparations did show a depolarization upon blue light exposure (Fig 3B1; N = 6, P<0.05 sign test). Also, as expected the yellow light activation of eNpHR resulted in a rapid hyperpolarization of the muscle cell in which it was expressed (Fig 3A3). However, the rapid onset was also followed by a rapid offset in tight synchrony with the light pulse. The reasoning of the flickering in the membrane potential during the light pulse is unknown. Possibly the motor nerve produces some intrinsic firing as it is still centrally connected or the eNpHR pump temporarily stops. The three repeated exposures of eNpHR with 10 minute dark adaptation produced varied responses and varied degrees of hyperpolarization (Fig 3B2). A hyperpolarization was evident in all six preparations examined (N = 6, P<0.05 sign test).

As expected, when the muscle fiber was depolarized with activation of ChR2-XXL (15 sec) and the motor nerve was electrically stimulated on the ipsilateral side to the NMJs being recorded, the evoked EPSPs were reduced in amplitude (Fig 3C1 and 3C2). The decrease in the amplitude of the evoked EPSPs was consistent in each preparation examined (Fig 3D1; N = 6, P<0.05 sign test). When the muscle was hyperpolarized with activation of the eNpHR pump (15 sec) during evoked EPSPs they increased in absolute amplitude (Fig 3C3). This enhanced EPSP amplitude was present in each preparation examined (Fig 3D2; N = 6, P<0.05 sign test). As for when the nerve is not stimulated, the prolonged depolarization with activation of ChR2-XXL in the muscle was present which presented a prolonged reduction in the EPSP amplitude. The smaller EPSP amplitude is due to decreased driving gradient. Likewise, with the increased driving gradient in the muscle fiber, the EPSPs are increased. Since the resting membrane returned quickly the effect on the EPSP returns rapidly to amplitudes prior to the eNpHR pump activation.

### Spontaneous quantal responses

**Fig 4:**
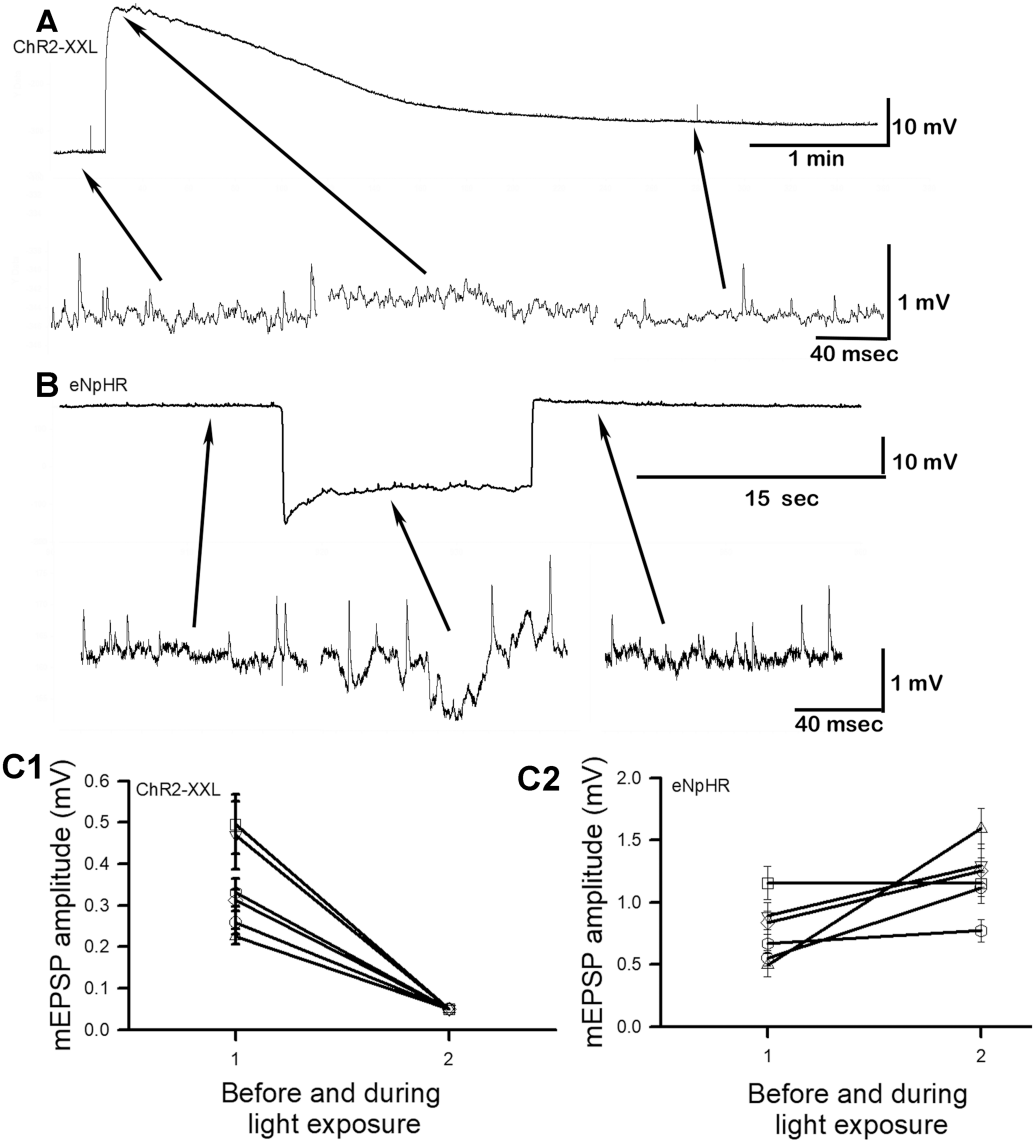
The activation of ChR2-XXL or eNpHR expressed in muscle fibers while recording spontaneous single quantal events (mEPSPs). (A) Exciting ChR2-XXL expressed in muscle with blue light depolarizes the muscle fibers resulting in smaller mEPSPs until the muscle can regain the resting membrane potential. (B) When eNpHR is expressed in muscle and activated with yellow light the muscle fibers hyperpolarize resulting in larger mEPSPs during the light pulse. Arrows indicate the times during the low gain recording where the traces for the mEPSPs are highlighted with higher resolution. The average amplitude (mean+/-SEM) of at least 10 mEPSPs events before and during light exposure for ChR2-XXL (C1) and eNpHR (C2) are shown.

Background spontaneous activity is recognized to be important for development and maintenance of synaptic transmission [46,66]. Since the dendrites of the motor neurons are not feasible to record synaptic responses electrophysiologically, we used the muscle fibers as a proof of concept to address the effects on quantal vesicular events. As with evoked responses, it is not surprising that the spontaneous single quantal events (mEPSP) mimic the same changes in amplitude as the driving gradients since the same receptor subtypes are activated. As the muscle is depolarized the mEPSPs were barely observable in the baseline noise (Fig 4A). This trend was consistent in each the six preparations examined in which at least an average of ten spontaneous quantal responses were measure before and during the peak of the depolarization (Fig 4C1; N = 6, P<0.05 sign test). The quantal spontaneous responses began to be appear again as the resting membrane potential was reestablished (Fig 4A, far right arrow). When the synaptic target cell is hyperpolarized the mEPSPs were readily apparent due to the larger amplitude (Fig 4B). Obtaining an average of at least ten spontaneous quantal events before and during the hyperpolarization illustrated the trend and variation among preparations (Fig 4C2).

## Discussion

In this brief report we highlighted an unexpected excitatory response within a neural circuit with the use of halorhodopsin (eNpHR). This light activated protein is commonly used to inhibit neural activity in the neurons in which it is expressed [25,27]. In the *Drosophila* larvae, when motor neurons are targeted to express eNpHR and during eNpHR activation, with yellow light, the neurons increased their response to excitatory synaptic activity within a functional neural circuit. The behavioral responses of intact larvae support the electrophysiological findings in that when the larvae are contracted during eNpHR activation in motor neurons there can be an increase in sensitivity to sensory drive. The motor neurons expressing ChR2-XXL also show prolonged sensitivity to sensory drive after the activation of ChR2-XXL is terminated. This excitation of ChR2-XXL can also lead to inhibition of the motor neuron depending on the duration and intensity of inducing CHr2-XXL [34].

To ensure eNpHR and ChR2-XXL were functioning as expected in our lines, we expressed the proteins in body wall muscle so direct electrophysiological measures could be obtained. In addition, the proof of concept in how eNpHR might lead to excitation of the motor neurons could be examined with evoked synaptic activity as well as spontaneous quantal events. The m6 muscle fiber has a much larger surface area than the neurons and potentially an enhanced synaptic efficacy than the dendrites of the motor neurons; however, the larger input resistance of the dendrites may also amplify the synaptic responses. The electrical responses measured in the muscle fibers with synaptic activity and channel activation for eNpHR and ChR2-XXL support the notion that the motor neurons have an increased responsiveness to synaptic activity by altering the driving gradients for excitatory synaptic activity. Likely the motor neuron would show different responses to the onset of eNpHR and CHR2-XXL stimulation and to the after effect when the stimulating light is turned off. There would be differences in the expression level and potentially the location of insertion within the membrane of the proteins in dendrites as compared to the muscle. In addition, the density and types of ion exchangers, pumps, leak channels and types of synaptic receptors would likely vary on dendrites and muscle fibers. The rapid changes in the muscle when eNpHR is activated were not observed with evoked the sensory-CNS-motor unit or with the whole animal behaviors. This is likely due to the isolated NMJ not integrating multiple synaptic inputs and having a variation in location of the induced expression of light sensitive channels in the dendrites, soma, axon and synaptic terminals as for neurons.

In rodents in which eNpHR is targeted to motor neurons by using a choline acetyltransferase construct (ChAT-eNpHR) a transient increase in the firing frequency of motoneurons was observed when the light was turned off [67]. This heightened response lasted more than 10 sec. The authors postulated this may have been due to a homeostatic compensation by a central pattern generator. However, when NBQX was used to block AMPA (ie. glutamate) receptors within the motor neuron circuit the enhanced firing frequency was not present. Inada et al., [39] noted that behaviorally there is inhibition of larval muscle contraction in *Drosophila* when eNpHR expressed in motor neurons was activated. However, upon turning the light off sometimes a contraction occurred. The electrophysiological measures we have shown may account for the potential of post-inhibitory rebound of the motor neurons which was observed in causing the contraction. Altering the ionic driving gradients of synaptic responses while eNpHR is activated may result in altered threshold of firing. This is worthy of further investigation in this preparation and others. An excitation phenomenon was also demonstrated in motor neurons of freshly hatched *Drosophila* larva by Giachello and Baines [68]. Their study suggested an anode break for the excitation and likely the removal of voltage gated inactivation which may occur in part at resting potentials. The enhanced drive of the sensory to CNS to motor circuit, with activation of eNpHR in motor neurons, we have shown supports this notion. It is very likely the same scenario is occurring in motor neurons for the 3^rd^ instar as we show indirectly with modeling in the muscle. We also illustrated the enhanced driving gradients for minis as well as the evoked responses while hyperpolarizing the cell with activation of eNpHR. In rodents, when inducing a penicillin epilepsy bout and trying to suppress the thalamus with eNpHR activation the duration of epileptiform bursts did not increase or decrease [69]. Such results suggest either the thalamic circuit is not involved in the circuit or that the neurons may not have been truly inhibited as anticipated with activating the Cl^-^ pump. Where membrane potentials are recorded with activation of eNpHR, depression of the evoked responses does occur in rodent hippocampal neurons [26] and with depolarized induced firing in cortical neurons [70].

**Fig 5:**
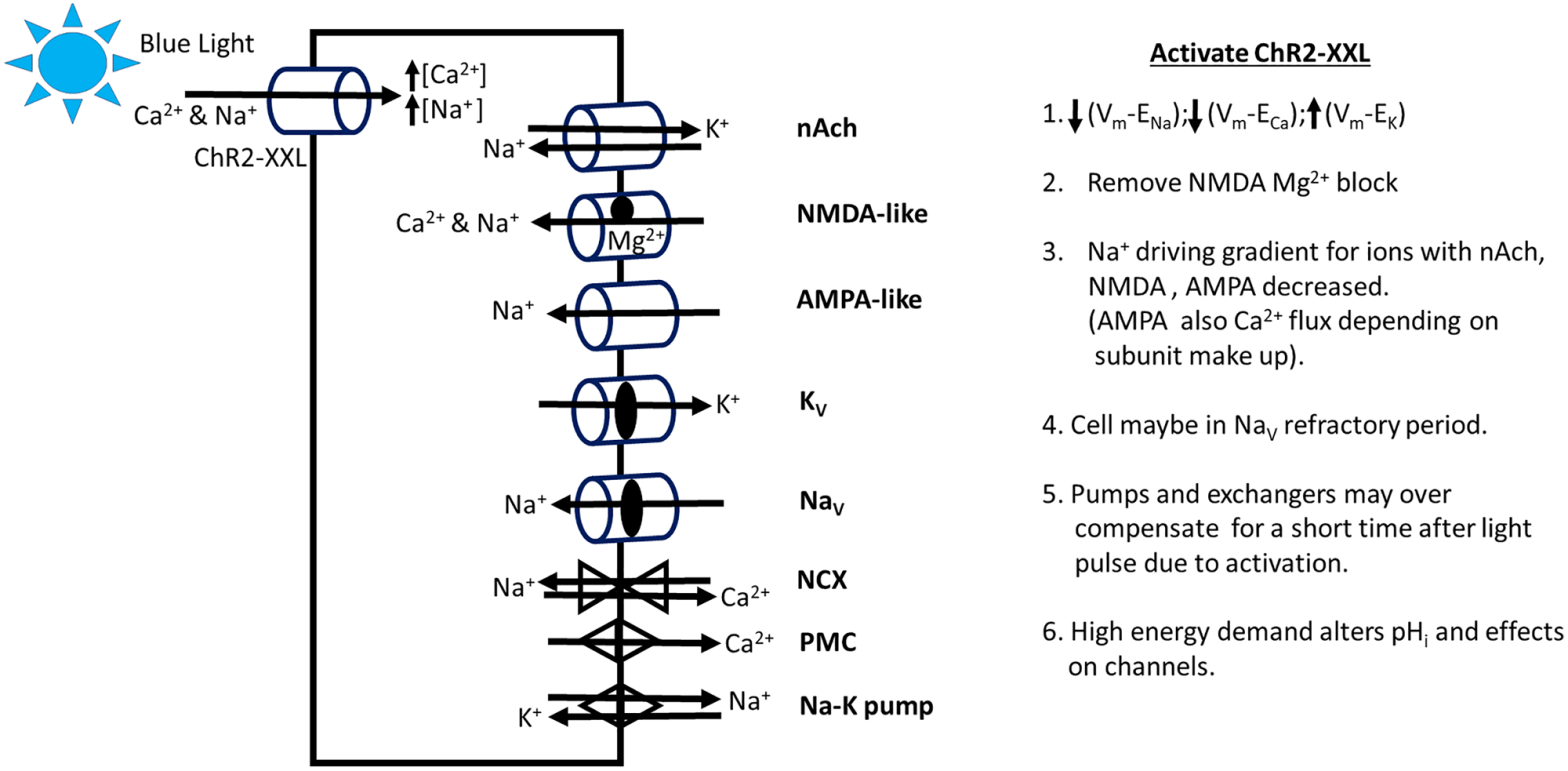
A theoretical model of the potential ionic responses in the motor neurons by activating the CHR2-XXL channel with blue light during synaptic transmission by either cholinergic or glutamatergic inputs. A partial list is provided of reactions to loading the neuron with Na^+^ and Ca^2+^ during the flux through CHR2-XXL. Potential receptors, ionic channels, exchangers and pumps present on the dendrites of motor neurons: nicotinic cholinergic (nAch), NMDA-like or AMPA-like glutamatergic receptors, voltage gated K^+^ (K_V_) and Na^+^ (Na_V_) channels, Na-Ca exchanger (NCX), plasma membrane Ca^2+^ pump (PMC) and the Na-K ATPase pump.

In attempt to address the potentially varied differences and postulate mechanisms responsible for the measured results two models are presented. One model outlines the cellular response for synaptic activity on a cell in which CHR2-XXL is activated resulting in a depolarized state due to Na^+^ and Ca^2+^ influx [71,72] (Fig 5). For either Ach or glutamate receptor mediated ionic conductance a depolarized state would dampen the driving gradients. However, if a NMDA-like receptor is expressed on the dendrites of motor neurons the reduction of the Mg^2+^ block might enhance a Ca^2+^ influx. A light induced depolarized state may activate voltage gated Ca^2+^, Na^+^ and K^+^ ionic channels but it would be expected that the Na^+^ channels would inactivate with maintained depolarization. This does appear to be a problem with too much activation of CHR2-XXL [33,34]. The electrogenic Na-K pump, plasma membrane calcium ATPase pump (PMCA) and exchangers would aid to reset the membrane potential after the light activation has ceased. The distribution of the channels, pumps and exchangers in the dendrites as compared to the rest of the neuronal membrane would determine the mix in the responses. In this simplistic model 2^nd^ messenger cascades are not highlighted but one should consider a myriad of potential consequences and the potential of the light activated channels being expressed in the membrane of the organelles resulting in ion fluxes.

**Fig 6:**
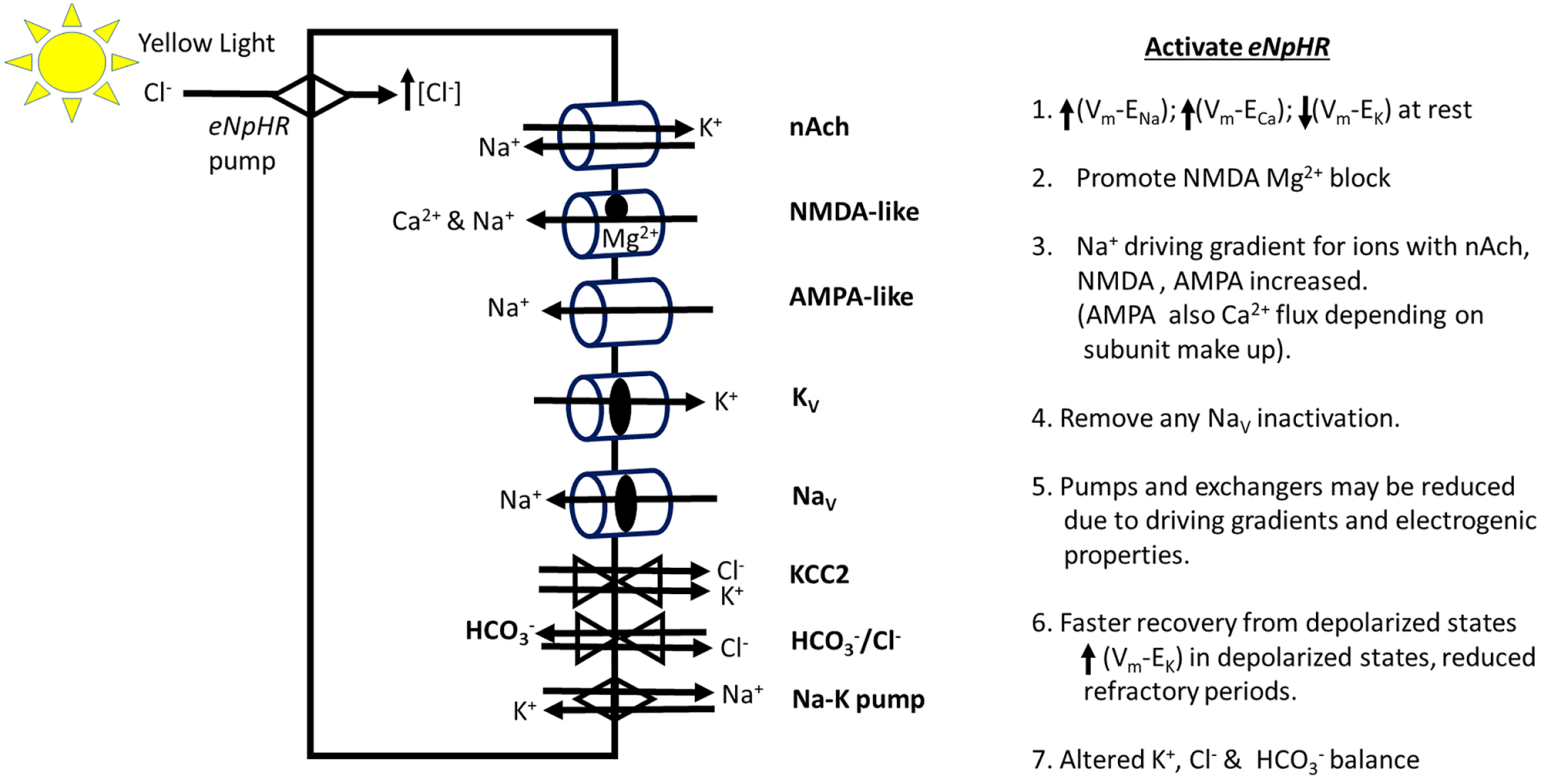
A theoretical model of the potential ionic responses in the motor neurons by activating the eNpHR chloride pump with yellow light during synaptic transmission by either cholinergic or glutamatergic inputs. A partial list is provided of reactions to loading the neuron with Cl^-^ during the flux. Potential receptors, ionic channels, exchangers and pumps present on the dendrites of motor neurons: nicotinic cholinergic (nAch), NMDA-like or AMPA-like glutamatergic receptors, voltage gated K^+^ (K_V_) and Na^+^ (Na_V_) channels, potassium chloride cotransporter 2 (KCC2), HCO_3_^-^/Cl^-^ exchanger (HCO_3_^-^/Cl^-^) and the Na-K ATPase pump.

The second model highlights the potential cellular response for synaptic activity when the chloride pump (eNpHR) is active during synaptic activity (Fig 6). With hyperpolarization the ionic driving gradient is enhanced for excitatory synaptic activity. However, if NMDA-like receptor is present potentially the Mg^2+^ block would be enhanced. The likelihood of reaching the threshold for activating voltage gated Ca2+, Na^+^ and K^+^ ionic channels would be decreased. However, if the resting cell shows some voltage gated Na^+^ channel inactivation then hyperpolarization can remove the inactivation resulting in a lowered threshold for activating the voltage gated Na+ channel. Thus, upon returning to the resting potential after the light induce eNpHR pump is turned off the cell may produce action potentials which normally would not have occurred [41,43–45]. However, one might assume this would be short lived after a few action potentials but the increased sensitivity to sensory drive in the intact neural circuit showed a prolonged sensitivity after the cessation of the light exposure. One possibility may be an enhanced activation of the KCC2 or HCO_3_^-^/Cl^-^ exchangers potentially producing an over compensation from the Cl^-^ which was pumped into the cell when the eNpHR was activated. This still does not seem too likely as there is not a precedent for prolonged over compensation from the Cl^-^ flux. Considering the membrane potential quickly resets to the resting state upon stopping the light activation eNpHR without a depolarization as would be expected if Cl^-^ was still being removed argues against this being the result of increased sensitivity to synaptic activity. If the eNpHR perhaps was incorporated in the ER and resulted in hyperpolarization this could result in ER stress and lead to a rise in intracellular Ca^2+^ [73]. However, high expression in mammalian neurons with eNpHR (a modified form of NpHR) was shown not to accumulate in organelles [74]. We are not aware if a similar study has been conducted for *Drosophila* neurons with the UAS-GAL4 expression system. Much work remains to resolve the mechanisms behind the unexpected prolonged sensitivity to synaptic drive in the neurons when eNpHR was activated. The acute responses might be readily rationalized by the ionic driving gradients being enhanced.

Given that the degree of sensitivity was not consistent to an evoked sensory-CNS-motor circuit when eNpHR was activated in the motor neurons, we could speculate this may be due to the various compensatory actions within each cell to the extent of activating the motor neurons by electrically stimulating the sensory roots. Although a minimal response was induced in the motor neurons with sensory activation prior to the light exposure of eNpHR. We do not think the neural circuit has compensated for inhibition of the motor neuron by background excitation of eNpHR as the larvae were maintained in the dark from 1st instars and while consuming the ATR from 2^nd^ to 3^rd^ instar prior to testing.

The results of hyperpolarizing the targeted postsynaptic neuron with a chloride channel would likely produce a different outcome than with the eNpHR chloride pump. An open Cl^-^ channel would rapidly suppress any depolarization back to the equilibrium potential of the Cl^-^ ion. Thus, a repeat of this study herein with the Cl^-^ channel (GtACR) is warranted [75,76]. An additional benefit would be to have a Ca^2+^ indicator and co-expressed with the CHR2-XXL or eNpHR (or GtACR) to determine the rates of buffering the intracellular Ca^2+^ concentrations and the fluxes during light induced channel activation. The current attempts in localizing the light sensitive proteins to regions of cells and their organelles will greatly advance the field of optically controlling cells with light [77,78]. Long-term, repetitive and reproducible control of neural circuits is a goal for the use of optogenetics without the unwanted side effects [38].

## Supporting information

S1 Video Larvae expressing ChR2-XXL in muscle are crawling around on the dish when exposed to infra-red light and upon blue light rapidly contract and remain contracted for some time after the blue light is stopped. (MP4)

S2 Video When eNpHR is expressed in motor neurons and exposed to yellow light for 15 sec the larvae become flaccid for a short time and then start to crawl. After dark exposure under infa-red the larvae regain full movement after some time.
(MP4)

S3 Video When eNpHR is expressed in muscle and exposed to yellow light for 15 sec the larvae show slight decreases in body wall locomotion and regain movement rapidly after the yellow light is turned off. (MP4)
